# tRNA Processing and Subcellular Trafficking Proteins Multitask in Pathways for Other RNAs

**DOI:** 10.3389/fgene.2019.00096

**Published:** 2019-02-20

**Authors:** Anita K. Hopper, Regina T. Nostramo

**Affiliations:** Department of Molecular Genetics, Center for RNA Biology, Ohio State University, Columbus, OH, United States

**Keywords:** tRNA, nuclear export, nuclear import, RNA processing, tRNA splicing

## Abstract

This article focuses upon gene products that are involved in tRNA biology, with particular emphasis upon post-transcriptional RNA processing and nuclear-cytoplasmic subcellular trafficking. Rather than analyzing these proteins solely from a tRNA perspective, we explore the many overlapping functions of the processing enzymes and proteins involved in subcellular traffic. Remarkably, there are numerous examples of conserved gene products and RNP complexes involved in tRNA biology that multitask in a similar fashion in the production and/or subcellular trafficking of other RNAs, including small structured RNAs such as snRNA, snoRNA, 5S RNA, telomerase RNA, and SRP RNA as well as larger unstructured RNAs such as mRNAs and RNA-protein complexes such as ribosomes. Here, we provide examples of steps in tRNA biology that are shared with other RNAs including those catalyzed by enzymes functioning in 5′ end-processing, pseudoU nucleoside modification, and intron splicing as well as steps regulated by proteins functioning in subcellular trafficking. Such multitasking highlights the clever mechanisms cells employ for maximizing their genomes.

## Introduction

**Figure 1:**
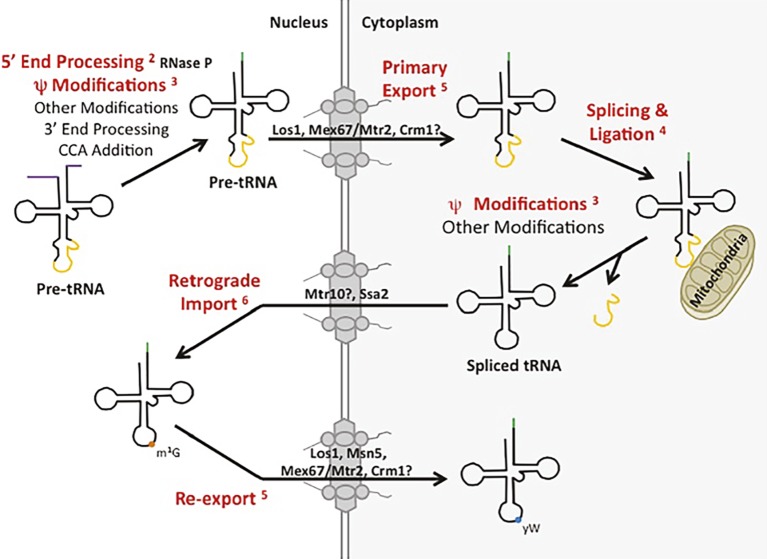
tRNA biogenesis and subcellular trafficking steps multitask in the processing/localization of other RNAs. Yeast tRNAs are synthesized as primary tRNAs (pre-tRNA) in the nucleolus and undergo subsequent 5′ and 3′ end processing, modifications (such as pseudouridine modification) and CCA addition, generating pre-tRNAs. These pre-tRNAs are then exported from the nucleus to the cytoplasm (shaded white and grey, respectively) *via* Los1, Mex67-Mtr2, or potentially Crm1 in a step termed tRNA primary nuclear export. tRNAs containing an intron are then spliced at the surface of the mitochondria by the SEN complex and the two tRNA halves are ligated by Rlg1/Trl1. Further nucleoside modifications also take place in the cytoplasm. Spliced tRNAs undergo a second trafficking step termed retrograde nuclear import, mediated by Ssa2 and potentially Mtr10. Once back in the nucleus, spliced tRNAs can be modified by enzymes that only recognize spliced tRNAs, and not intron-containing tRNAs, such as Trm5-catalyzed methylation of G at position 37 (m^1^G_37_; orange circle). tRNAs are then re-exported from the nucleus to the cytoplasm by any of the primary exporters or Msn5, which functions solely in the re-export step, to be utilized in translation. Certain tRNAs receive additional modifications, such as modification of the m^1^G_37_ to wybutosine (yW; blue circle) in tRNA^Phe^ in yeast. The tRNA biogenesis and subcellular trafficking steps highlighted in red indicate pathways utilized by not only tRNA, but other RNA species as well. Each is discussed in the text and the appropriate figure numbers are indicated in superscript.

Biogenesis of different categories of eukaryotic RNAs has been thought to proceed *via* distinct pathways. Indeed, eukaryotic cells employ separate DNA-dependent RNA polymerases, Pol I, II, and III, to transcribe precursors to ribosomal RNA (pre-rRNA), mRNA (pre-mRNA), and tRNA (pre-tRNA), respectively. Moreover, the cell biology for processing the various categories of pre-RNAs appears to be quite different. For example, mRNA splicing, capping, polyadenylation, and nuclear export all occur co-transcriptionally [Review: (Bentley, 2014)]. In contrast, tRNA biogenesis occurs post-transcriptionally at numerous distinct subcellular locations. In *S. cerevisiae* (budding yeast), pre-tRNA transcription by Pol III and 5′ maturation, catalyzed by RNase P, are located in the nucleolus [Review: (Hopper et al., 2010)], whereas particular tRNA modifications are added in the nucleoplasm, and other modifications are added at the inner nuclear membrane or in the cytoplasm after tRNA nuclear export [Review: (Hopper, 2013)]; moreover, pre-tRNA splicing occurs on the surface of mitochondria (Yoshihisa et al., 2003) (Figure 1). Despite the general requirement for separate DNA-dependent RNA polymerases and the different subcellular locations of major processing events, it is now appreciated that particular RNA processing/modification enzymes and pathways for intracellular dynamics can be shared among distinct RNA categories to facilitate related, if not identical, functions. Here, we detail well-established examples (Figure 1, example steps in red font) of proteins and RNP complexes involved in tRNA biology that also function in the biogenesis and subcellular trafficking of other categories of RNAs, RNPs, and proteins.

### Shared Subunits for RNPs Involved in 5′ Pre-tRNA Processing, Pre-rRNA Processing, and Telomerase

**Figure 2:**
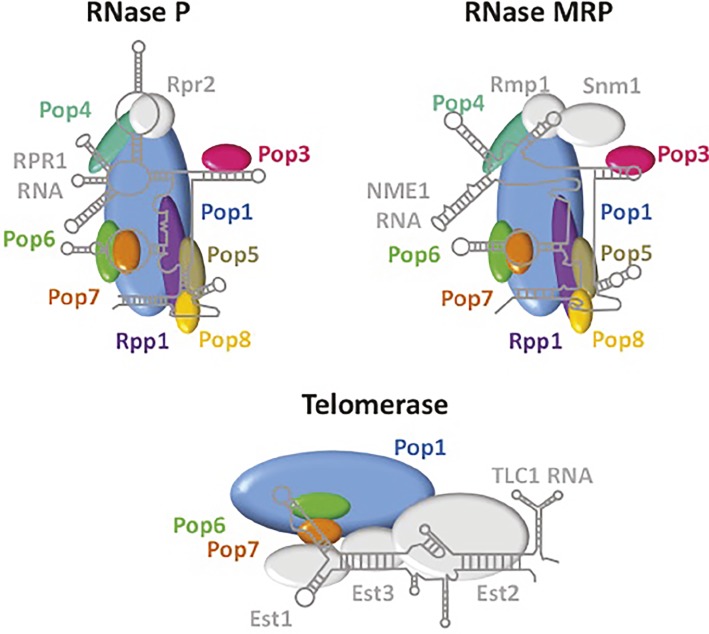
RNase P, RNase MRP, and telomerase share protein subunits. Depicted are the RNA and proteins that comprise the RNPs of RNase P, RNase MRP and telomerase. Pop 1 (blue), Pop6 (green), and Pop7 (orange) are shared among all three RNPs, whereas other proteins are shared only between RNase P and RNase MRP (Pop3, pink; Pop4, teal; Pop5, brown; Pop8, yellow; Rpp1, purple). RNAs and proteins that are unique among each of these complexes are indicated in grey. The structures of RNA and protein components are derived from Lemieux et al. (2016) for telomerase and from Gopalan et al. (2018) for RNase P. The structure of RNase MRP was extrapolated from the RNase P structure.

RNase P is an endonuclease that removes 5′ leaders from pre-tRNAs (Figure 1). It is a ribonuclear protein (RNP) complex in bacteria, many archaea, and in the nucleus of budding yeast and metazoans; in these organisms, RNase P is comprised of a single small catalytic RNA and various numbers of proteins [Review: (Gopalan et al., 2018)]. Surprisingly, RNase P is a protein-only enzyme (PRORP) in plants and in the organelles of various organisms (Gutmann et al., 2012). In budding yeast, the RNase P complex that processes nucleus-encoded tRNAs is located in the nucleolus and the complex consists of RPR1, the RNA subunit, and nine essential proteins, Pop1, Pop3, Pop4, Pop5, Pop6, Pop7, Pop8, Rpp1, and Rpr2 [Review: (Xiao et al., 2002)]. Unexpectedly, RNase P shares protein subunits with RNPs that have different functions (Figure 2). All RNase P proteins except Rpr2 and the RPR1 RNA are shared with MRP, a nucleolar RNP that functions in the processing of pre-rRNA (Xiao et al., 2002; Lindahl et al., 2009; Gopalan et al., 2018). Moreover, Pop1, Pop6, and Pop7 are also components of telomerase, the RNP that is required for the replication of chromosome termini (Lemieux et al., 2016). So, several proteins of the RNase P RNP multitask in at least three separate processes. Structural studies have delineated how the various proteins of these three independent RNPs interact with their unshared RNA subunit (Figure 2), but interesting and important questions remain regarding the evolutionary selection for sharing of subunits among these RNPs with quite different functions.

### Numerous RNA Substrates for the “tRNA” Pseudouridine Synthetases

**Figure 3:**
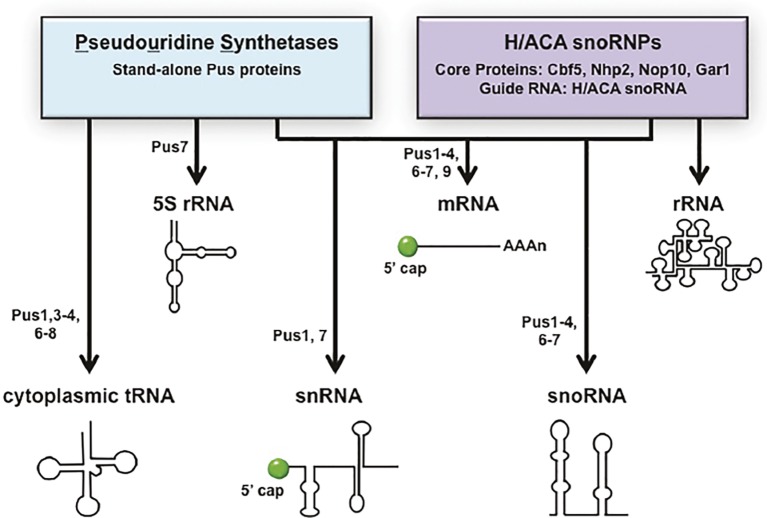
The “tRNA” pseudouridine synthetases also modify mRNA, snRNAs, and snoRNAs. Pseudouridine modification of various RNAs is catalyzed by stand-alone proteins called pseudouridine synthetases (Pus) or RNPs containing guide RNAs called H/ACA snoRNPs. tRNAs and 5S RNA are pseudouridinylated solely by Pus proteins and rRNAs solely by H/ACA snoRNPs. Pseudouridine modifications of mRNA, snRNA, and snoRNA are catalyzed by either Pus proteins or H/ACA snoRNPs in a site-specific manner. The specific Pus proteins known to pseudouridinylate each RNA species are indicated. RNA substrates are not drawn to scale.

Throughout the steps of pre-tRNA maturation, tRNAs acquire nucleoside modifications. tRNAs are highly modified. There are >100 nucleotide modifications known to occur on tRNAs [Reviews: (Phizicky and Hopper, 2010; Jackman and Alfonzo, 2013)]. Budding yeast has 25 different tRNA modifications, and each mature tRNA contains an average of ~12 modified nucleosides. Nearly all the genes encoding proteins required for tRNA modification in budding yeast have been identified and characterized (Phizicky and Hopper, 2010; Hopper, 2013). Pseudouridine (ψ) is one of the most abundant of the tRNA modifications. The enzymes that catalyze pseudouridylation of nucleus-encoded cytoplasmic tRNAs (Pus1, Pus3, Pus4, Pus6, Pus7, and Pus8) are protein enzymes (Phizicky and Hopper, 2010). Ribosomal 5S RNA, small nuclear RNAs (snRNA) that are mRNA splicing components, and small nucleolar RNAs (snoRNA) that function in pre-rRNA processing, and rRNAs all also contain pseudouridine modifications. 5S rRNA ψ modification is catalyzed by Pus7 in budding yeast (Decatur and Schnare, 2008). Pseudouridine modifications of snRNA and snoRNA are more complicated as, in addition to ψ modifications being catalyzed by particular Pus proteins, they are also catalyzed by H/ACA pseudouridylases. H/ACA pseudouridylases are RNA-dependent RNP complexes with guide RNAs (yeast H/ACA RNAs). In budding yeast, ψ modifications of snRNAs are catalyzed by Pus1 or Pus7 or by RNA-dependent RNP enzymes, and snoRNAs ψ modifications are catalyzed by Pus1–4 and Pus6,7 in addition to H/ACA pseudouridylases. In contrast, rRNAs pseudouridylation is catalyzed solely by H/ACA pseudouridylases [Reviews: (Karijolich and Yu, 2010; Rintala-Dempsey and Kothe, 2017)] (Figure 3).

New genome-wide technologies have led to the discovery that mRNAs also contain pseudouridine modifications and that the mRNA modifications are generated by both Pus proteins and the H/ACA RNP. Scores of budding yeast mRNAs are modified by the Pus proteins and each of the *PUS* genes that encode nuclear or cytoplasmic enzymes contributes to mRNA modifications at particular sites. Furthermore, some mRNA sites are modified only under particular stress conditions [(Carlile et al., 2014; Schwartz et al., 2014) Reviews: (Gilbert et al., 2016; Rintala-Dempsey and Kothe, 2017)]. Thus, the protein “tRNA” pseudouridylases multitask in the biogenesis of tRNAs, 5S RNA, snRNAs, snoRNAs, and mRNAs (Figure 3).

### Multitasking by Pre-tRNA Splicing Enzymes

In addition to 5′ and 3′ end-processing and addition of nucleoside modifications, some pre-tRNAs contain transcribed short introns, located one nucleotide 3′ to the anticodon, that must be removed to generate functional tRNAs. All known eukaryotic genomes encode a subset of intron-containing tRNA genes, although the percentage of such genes varies considerably (Chatterjee et al., 2018). Pre-tRNA splicing is essential in most eukaryotes because generally all reiterated copies of at least one particular isoaccepting tRNA family are encoded by intron-containing genes. The tRNA splicing process is catalyzed endonucleolytic cleavage that generates an intron and two exons, each about half the size of the mature tRNA, followed by ligation of the two resulting exons.

#### Splicing Endonuclease Complex Cleaves mRNAs in Addition to Pre-tRNAs

**Figure 4:**
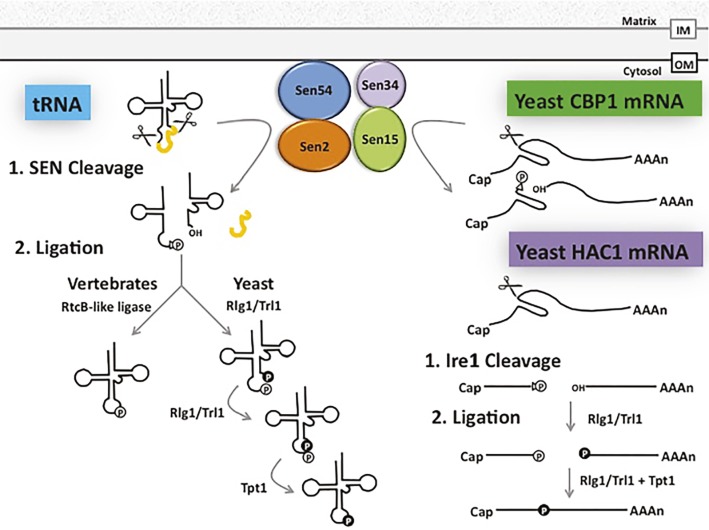
Shared pathways for splicing and ligation of tRNA and mRNA. tRNA introns (depicted in yellow) are removed by the heterotetrameric SEN complex at the outer surface of the mitochondrial membrane in budding and fission yeast, generating a 2′-3’cyclic phosphate (triangle and “P” in white circle) at the 3′ end of the 5′ exon and a hydroxyl group at the 5′ end of the 3′ exon. In budding yeast, the tRNA 5′ and 3′ exons are ligated by Rlg1/Trl1 in the cytoplasm in a series of reactions beginning with the phosphorylation of the 5′ hydroxyl group (“P” in black circle) of the 3′ exon and decyclization of the 2′-3′ cyclic phosphate to a 2′ phosphate (“P” in white circle) on the 5′ exon. These exons are then joined using the 5′ phosphate, leaving the 2′ phosphate at the splice site to be transferred to NAD by Tpt1. In vertebrates, however, the vertebrate ligase directly joins the 2′-3′ cyclic phosphate of the 5′ exon to the 3′ exon. The SEN complex also functions in the clipping of CBP1 mRNA and Rlg1/Trl1 ligates HAC1 mRNA exons generated by a protein endonuclease. Additional, but currently unidentified, RNAs are likely also processed by the SEN complex (see text). OM: Outer mitochondrial membrane, IM: Inner mitochondrial membrane.

Introns in yeast and vertebrate pre-tRNAs are removed by the conserved heterotetrameric splicing endonuclease (SEN) (Trotta et al., 1997; Paushkin et al., 2004) (Figure 4). SEN is located on the cytoplasmic surface of mitochondria in budding and fission yeast, but it is in the nucleoplasm in *Xenopus* oocytes and HeLa cells (De Robertis et al., 1981; Yoshihisa et al., 2003, 2007; Paushkin et al., 2004; Wan and Hopper, 2018). The SEN complex fails to cleave tRNAs that possess inappropriately structured mature domains, and it will cut pre-tRNAs at inappropriate sites if the length of the anticodon stem is altered [(Reyes and Abelson, 1988) Review: (Yoshihisa, 2014)]. Although, to date, there is not a high-resolution co-structure of SEN with its pre-tRNA substrate, the data support the model that SEN interacts with the mature tRNA 3d structure and it “measures” the length of the anticodon stem. Therefore, reports documenting that non-tRNAs also serve as SEN substrates were surprising. The SEN complex has been reported to cleave an mRNA that encodes the unessential cytochrome b mRNA processing 1 protein, Cbp1 (Tsuboi et al., 2015) (Figure 4). Moreover, other studies showed that mitochondrial-located SEN catalytic activity in budding yeast is essential even when cells generate spliced tRNAs *via* alternate mechanisms (Dhungel and Hopper, 2012; Cherry et al., 2018), implicating the SEN complex in processing of additional essential RNAs. However, the essential non-tRNA substrate(s) remain unknown. Thus, SEN has the capacity to cleave mRNAs and perhaps other unidentified RNAs in addition to its essential and well-studied role in catalyzing intron removal from pre-tRNAs (Figure 4).

#### “tRNA” Splicing Ligases have mRNA Substrates

There are two evolutionarily distinct mechanisms to ligate tRNA exons. Surprisingly, for both mechanisms, the ligase that joins pre-tRNA exons after cleavage also ligates a non-tRNA substrate. Pre-tRNA intron removal by all SEN complexes generates a 5′ exon bearing a 2′,3′ cyclic phosphate and a 3′ exon with a 5′ hydroxyl (Knapp et al., 1979; Peebles et al., 1983). In yeast and plants, ligation of the two tRNA exons is catalyzed by Rlg1/Trl1, that has 2′,3′ cyclic phosphodiesterase, 5’ RNA kinase, and RNA ligase activities (Phizicky et al., 1986; Englert and Beier, 2005; Wang and Shuman, 2005). Ligation by Rlg1/Trl1 generates a splice junction with two phosphates; the extra 2′ phosphate is removed *via* a transferase activity catalyzed by Tpt1 (Culver et al., 1997).

Although the mechanism for tRNA exon ligation is similar in budding yeast and plants (Englert and Beier, 2005), the mechanisms for tRNA ligation are different in vertebrates and archaea. In vertebrates and archaea, tRNA ligation after removal of tRNA introns is catalyzed by an enzyme complex consisting of a RtcB-like ligase (Tanaka et al., 2011), HSPC117, that catalyzes direct joining of the cleaved 5′ exon bearing a 3′ phosphate (*via* 2′,3′ phosphodiesterase activity of the ligase) with the 5′ hydroxyl on the 3′ exon [(Popow et al., 2011) Review: (Popow et al., 2012)] (Figure 4).

Both the Rlg1/Trl1 and the RtcB-like ligases also catalyze exon joining of a particular mRNA. Rlg1/Trl1 catalyzes ligation of HAC1 exons generated *via* non-conventional cleavage by an endonuclease, Ire1, rather than by the spliceosomal mechanism (Sidrauski and Walter, 1997). Cleavage of HAC1 mRNA by Ire1 generates a 5′ exon possessing a 2′,3′ cyclic phosphate and a 3′ exon possessing a 5′ hydroxyl. Subsequent ligation of these exons by Rlg1/Trl1 is mechanistically identical to ligation of tRNA exons (Figure 4). Ligation of the exons thereby generates mature HAC1 mRNA that functions in the unfolded protein response (Sidrauski et al., 1996; Gonzalez et al., 1999). So, the tRNA ligase, Rlg1/Trl1, is, in fact, also a ligase for HAC1 mRNA. Similarly, plant bZIP60, which also functions in the unfolded protein response, is generated by nonconventional mRNA splicing. Plant IRE endonuclease cuts pre-bZIP60, generating two exons that are subsequently ligated by tRNA ligase, RLG1 (Nagashima et al., 2016). Thus, both SEN and ligase of budding yeast and plants participate in maturation of mRNA substrates in addition to tRNA substrates.

In *Xenopus* oocytes and HeLa cells, pre-tRNA splicing occurs in the nucleoplasm (De Robertis et al., 1981; Lund and Dahlberg, 1998; Paushkin et al., 2004). Therefore, vertebrate SEN and HSPC117 ligase must also be located in the nucleoplasm. However, there is also a cytoplasmic pool of HSPC117/RtcB that generates a spliced mature mRNA that encodes a protein which functions in the unfolded protein response. HSPC117/RtcB ligates XBP1 mRNA exons whose intron, like for HAC1, is removed in the cytoplasm by nuclease activity, rather than by the spliceosomal mechanism (Kosmaczewski et al., 2014). So, as for budding yeast and plants, vertebrate tRNA splicing ligase multitasks to ligate an mRNA in addition to its essential role in ligation of tRNA exons, even though the budding yeast and plant ligases are mechanistically different from the vertebrate ligase.

### Multitasking Nuclear Exporters/Importers for Nuclear-Cytoplasmic Dynamics of tRNAs As Well As Other RNAs, RNPs, and Proteins

In all eukaryotes, RNAs and proteins traffic between the nucleus and the cytoplasm. There are two distinct mechanisms by which macromolecules move between the nucleus and the cytoplasm. However, with the notable exception of most mRNAs, movement of macromolecules between the nucleus and cytoplasm generally employ the mechanism that depends upon the small GTPase, Ran, and the β-importin family of proteins [Reviews: (Gorlich and Kutay, 1999; Cook et al., 2007]. Those β-importin family members involved in import of macromolecules into the nucleus are termed importins and those functioning in export of macromolecules from the nucleus to the cytoplasm are termed exportins, although a few of the family members function both in nuclear import and export (Yoshida and Blobel, 2001; Aksu et al., 2018). The importins and exportins bind appropriate substrates, FG nuclear pore proteins, and Ran-GTP. Ran is primarily in the GTP-bound state in the nucleus and the GDP-bound state in the cytoplasm, thereby creating a Ran-GTP gradient between the nucleus and the cytoplasm. This Ran-GTP nuclear/cytoplasmic gradient determines the directionality of the movement of macromolecules between the nucleus and cytoplasm.

tRNAs move dynamically between the nucleus and the cytoplasm in yeast, protozoa, and vertebrate cells (Shaheen and Hopper, 2005; Takano et al., 2005; Shaheen et al., 2007; Huynh et al., 2010; Barhoom et al., 2011; Ohira and Suzuki, 2011; Watanabe et al., 2013; Dhakal et al., 2018; Kessler et al., 2018). Even though only a subset of eukaryotic tRNA-encoding genes contains introns, we focus on this category of tRNAs. This is because removal of introns from pre-tRNAs serves as a useful reporter for the steps of tRNA subcellular dynamics.

#### Primary tRNA Nuclear Export

Nuclear-cytoplasmic tRNA dynamics consists of three steps: primary nuclear export, retrograde tRNA nuclear import, and tRNA nuclear re-export (Chatterjee et al., 2018). Here, we first describe the proteins that function in primary tRNA nuclear export followed by a description of the proteins functioning in the remaining two trafficking steps (Figure 1). In budding and fission yeast, it is possible to distinguish between primary nuclear export and tRNA nuclear re-export because pre-tRNA splicing takes place on the surface of mitochondria (Yoshihisa et al., 2003, 2007; Wan and Hopper, 2018) and therefore, defects in primary nuclear export cause nuclear accumulation of unspliced pre-tRNAs. In contrast, tRNAs that have been exported to the cytoplasm *via* primary nuclear export and subsequently spliced prior to import back to the nucleus will accumulate spliced tRNA in the nucleus if the cells are defective in the re-export step. As detailed below, primary nuclear export and tRNA nuclear re-export can also be distinguished by assessing the status of the m^1^G_37_ and queuosine (Q_34_) nucleoside modifications in budding yeast and *T. brucei*, respectively.

#### Los1/Exportin-t/Xpot/PSD

**Figure 5:**
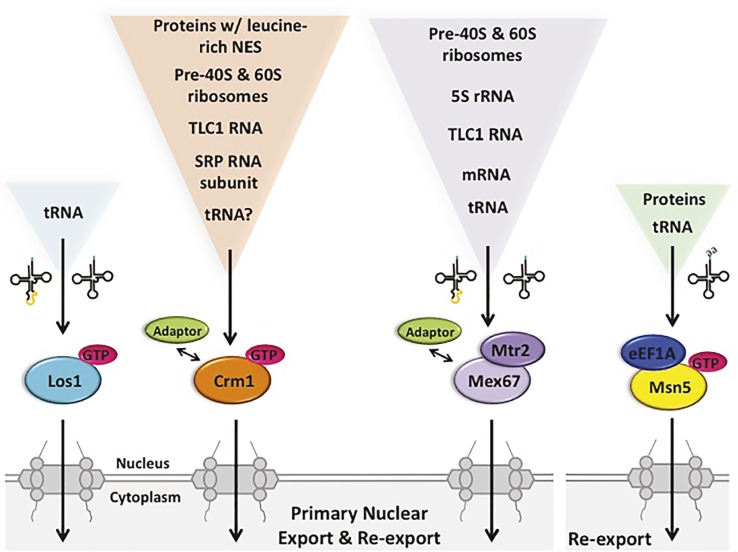
The multiple pathways for budding yeast primary tRNA nuclear export and re-export are also utilized for the export of other RNA and protein species. Primary export and re-export of tRNAs from the nucleus to the cytoplasm occurs *via* Ran-dependent and Ran-independent exporters, specifically Los1, Mex67-Mtr2 and possibly Crm1 (left), which interact with both spliced and unspliced tRNAs. Msn5, however, selectively re-exports charged tRNAs, forming a quaternary complex with Ran-GTP and eEF1A (right). Although Los1 is a dedicated tRNA nuclear exporter, Mex67-Mtr2, Msn5 and Crm1 can export various RNA and/or protein species with the assistance of different adaptor proteins. GTP, Ran-GTP; SRP, signal recognition particle; NES, nuclear export sequence.

One member of the β-importin family, Los1 (budding yeast)/Xpot (fission yeast)/Exportin-t (vertebrates)/PSD (plants), is dedicated to tRNA nuclear export (Hellmuth et al., 1998; Kutay et al., 1998; Sarkar and Hopper, 1998; Arts et al., 1998a). Los1/Exportin-t binds both intron-containing and intron-lacking tRNAs in a Ran-GTP-dependent fashion, and it does not require protein adaptors (Arts et al., 1998b; Lipowsky et al., 1999; Cook et al., 2009; Huang and Hopper, 2015). No other cellular RNAs have been reported to interact with Los1 or its various homologues. So, unlike the other proteins described in this review, it appears that Los1 and its homologues do not multitask in nuclear export of other RNAs or proteins (Figure 5).

Los1/Exportin-t is unessential in every tested organism, including budding yeast, fission yeast, plants, and haploid human cancer cells (Hurt et al., 1987; Hunter et al., 2003; Cherkasova et al., 2012; Blomen et al., 2015; Hart et al., 2015; Wang et al., 2015). Moreover, insects lack an Exportin-t homologue (Lippai et al., 2000). Since tRNAs must be efficiently delivered to the cytoplasm for their essential role in protein synthesis, other export pathways also function in tRNA nuclear export. Genome-wide studies with budding yeast identified candidate proteins that function in primary tRNA nuclear export (Wu et al., 2015): Crm1 (yeast)/Exportin 1 or Xpo1 (vertebrates) (Wu et al., 2015) and Mex67-Mtr2 (yeast)/NXF1-NXT1 or TAP-p15 (metazoans) (Wu et al., 2015; Chatterjee et al., 2017, 2018).

#### Crm1/Exportin-1 – A β-Importin That Functions in Nuclear Export of A Variety of Macromolecules

The β-importin family member Crm1/Exportin-1 functions in nuclear export of proteins possessing leucine-rich nuclear export sequences (NES) (Fischer et al., 1995; Wen et al., 1995). Crm1 also functions in nuclear export of several types of RNA *via* interactions with adaptor proteins possessing the leucine-rich NES. In budding yeast, Crm1 mediates nuclear export of the large and small precursor ribosomal subunits [(Sengupta et al., 2010) Reviews: (Okamura et al., 2015; Chaker-Margot, 2018)], the RNA subunit of signal recognition particle (SRP) (but not in vertebrate cells) (Grosshans et al., 2001; Takeiwa et al., 2015), and TLC1, the RNA subunit of telomerase [(Gallardo et al., 2008) Review: (Vasianovich and Wellinger, 2017)], *via* interactions of the cargo RNA/RNPs with adaptor proteins containing the leucine rich motif (Figure 5). In vertebrate cells, Exportin-1 also functions in nuclear export of snRNAs and particular mRNAs involved in stress and particular viral RNAs [Reviews: (Kohler and Hurt, 2007; Delaleau and Borden, 2015)].

Crm1 is also implicated in primary tRNA nuclear export because yeast cells possessing a temperature sensitive (ts) mutation of *CRM1* accumulate unspliced tRNA at the nuclear rim at the non-permissive temperature (Wu et al., 2015). Furthermore, Crm1 and Los1 genetically interact as *crm1–1 los1Δ* double mutants have synthetic growth defects (Wu et al., 2015). However, to date, there are no publications that document direct interactions between Crm1 and intron-containing tRNA; thus, Crm1 could mediate primary tRNA nuclear export indirectly. In summary, the exportin Crm1 is involved in nuclear export of numerous RNAs and RNPs, and perhaps also pre-tRNA nuclear export (Figure 5).

#### Mex67-Mtr2/NXF1-NXT1—A Ran-GTP Independent Mechanism for Nuclear Export of Numerous RNAs

Although it is well established that nuclear export of small structured RNAs like tRNAs, snRNAs, SRP RNA, and TLC1 as well as ribosomal subunits employ one or more members of the β-importin family, nuclear export of mRNAs is largely independent of the Ran-GTP mechanism [Reviews: (Kelly and Corbett, 2009; Okamura et al., 2015)]. Instead, mRNA nuclear export occurs *via* sequential rearrangements of multiprotein RNA-binding complexes. In metazoan cells, a transcription-dependent protein complex, TREX, is recruited near mRNA 5′ caps *via* interaction with the cap-binding complex and then a heterodimer, NXF1-NXT1 (TAP-p15) is recruited before nuclear export proceeds. The yeast heterodimeric homologue, Mex67-Mtr2, appears to interact with mRNAs *via* a transcription-dependent 3′ end processing mechanism using several protein adaptors (Kelly and Corbett, 2009). The Mex67-Mtr2 heterodimer and the metazoan homologues also function in nuclear export of other RNAs (Figure 5). They function in nuclear export of 5S rRNA (Yao et al., 2007), the pre-60S ribosome (Yao et al., 2007), the pre-40S ribosome (Faza et al., 2012), TLC1 RNA (Wu et al., 2014), and, in vertebrates, type D unspliced retroviral RNAs (Ernst et al., 1997; Pasquinelli et al., 1997).

Recently, Mex67-Mtr2 in budding yeast has been shown to export tRNAs to the cytoplasm (Chatterjee et al., 2017). Incubation of yeast harboring temperature-sensitive mutations of the essential *MEX67* and *MTR2* genes at a non-permissive temperature results in nuclear accumulation of end-processed, intron-containing tRNAs, similar to the phenotype of yeast lacking *LOS1* (Wu et al., 2015; Chatterjee et al., 2017). Moreover, providing yeast cells lacking Los1 (β-importin) with a mere fivefold excess of ectopic Mex67-Mtr2 results in efficient suppression of both phenotypes of *los1Δ*–accumulation of unspliced tRNAs and accumulation of tRNA in nuclei (Chatterjee et al., 2017). So, Mex67-Mtr2 is able to substitute for Los1, if cells are provided with sufficient quantities of the heterodimer. *In vivo* biochemical studies documented that protein A-tagged Mex67 co-purifies with intron-containing pre-tRNA as well as spliced tRNA (Chatterjee et al., 2017). The data support the model that Mex67-Mtr2 functions directly in tRNA nuclear export in both the primary and the re-export steps. It is unknown whether vertebrate cells employ Mex67-Mtr2 for tRNA nuclear export. However, NXT1 has been reported to stimulate nuclear tRNA export in permeabilized HeLa cells (Ossareh-Nazari et al., 2000) (Figure 5).

How Mex67-Mtr2 interacts with tRNAs remains unknown as none of the previously described Mex67-Mtr2 adaptors were uncovered in the genome-wide screen for tRNA splicing defects (Kelly and Corbett, 2009; Wu et al., 2015). Therefore, if Mex67-Mtr2 interaction with tRNA occurs *via* an adaptor, the adaptor may be encoded by redundant genes or it may be novel. It is also feasible that Mex67-Mtr2 interacts with tRNAs without employing an adaptor, similar to reported interactions of NXF1-NXT1 with particular heat shock mRNAs (Zander et al., 2016). It is also unknown what tRNA structures are important for interaction with Mex67-Mtr2.

In summary, there are at least two (i.e., Los1 and Mex67-Mtr2) and perhaps three (i.e., Crm1) or more, pathways in budding yeast for primary tRNA nuclear export. Both Mex67-Mtr2 and Crm1 are involved in the nuclear export of numerous other RNAs; so, unlike Los1, they are not dedicated to tRNA nuclear export (Figure 5). It will be interesting to learn whether they have the same fidelity for exporting tRNAs that are appropriately structured and processed to the cytoplasm as does Los1.

#### Retrograde tRNA Nuclear Import

The quandary that the budding yeast SEN complex is located on the surface of mitochondria but spliced tRNAs accumulate in the nucleus under particular stress conditions (Sarkar and Hopper, 1998; Grosshans et al., 2000; Azad et al., 2001; Feng and Hopper, 2002; Takano et al., 2005) led the Hopper and Yoshihisa labs to consider the unorthodox possibility that tRNAs in the cytoplasm could travel in a retrograde direction back to the nucleus. Employing RNA florescence *in situ* hybridization (FISH), these labs demonstrated that ectopic “foreign” tRNAs encoded by one nucleus of a heterokaryon could travel to and accumulate in the nucleus that did not encode the tRNA (Shaheen and Hopper, 2005; Takano et al., 2005), providing strong evidence for tRNA retrograde nuclear import (Figure 1). Other studies of haploid yeast and rat hepatoma cells in culture showed that tRNAs accumulate in nuclei upon nutrient deprivation even when transcription of new tRNAs is inhibited by thiolutin or actinomycin D, respectively (Takano et al., 2005; Shaheen et al., 2007; Whitney et al., 2007). Employing nuclear import assays with permeabilized HeLa cells, the Fassati group demonstrated that tRNA nuclear import occurs in vertebrate cells and their studies also showed that tRNA retrograde traffic provides one mechanism by which the retrotranscribed HIV genome can access the nuclear interior in nondividing cells (Zaitseva et al., 2006). Subsequent RNA FISH studies in protozoa, brine shrimp, and vertebrate cells in culture, and, most recently, tagged-tRNAs injected into vertebrate live cells, demonstrate widespread conservation of nuclear import of cytoplasmic tRNAs, especially in response to nutrient and/or heat stress [(Huynh et al., 2010; Barhoom et al., 2011; Miyagawa et al., 2012; Watanabe et al., 2013; Chen et al., 2016; Dhakal et al., 2018) Review: (Huang and Hopper, 2016)].

**Figure 6:**
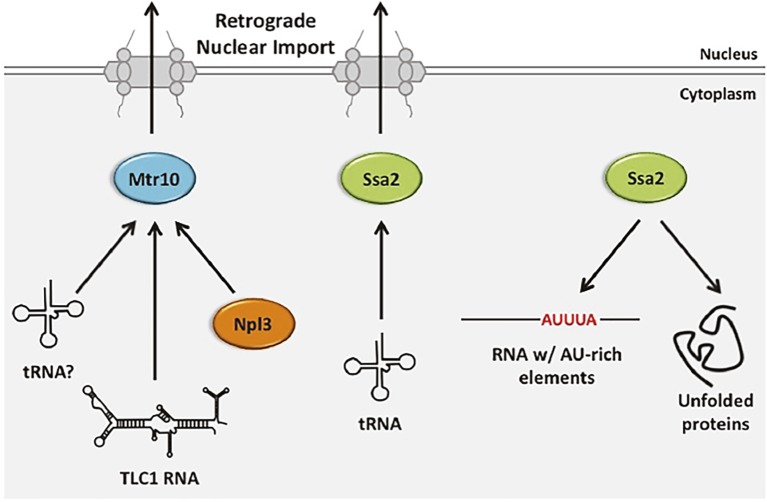
The role of budding yeast retrograde tRNA nuclear importers in nuclear import and gene expression regulation of other RNA and proteins. Retrograde tRNA nuclear import is mediated in a Ran-GTP-dependent manner, likely by Mtr10 and a Ran-GTP-independent manner by Ssa2. Mtr10 is also an importer of nuclear export protein Npl3 and it is implicated as a nuclear importer for the TLC1 RNA of telomerase. Ssa2 has additional non-import related functions, including its well-characterized role in protein folding. Members of the Hsp70 family also bind RNAs containing AU-rich elements, leading to their degradation.

The mechanism of tRNA retrograde nuclear import remains poorly understood. However, it is thought that tRNA retrograde nuclear import occurs constitutively as well as being upregulated in response to stress (Chatterjee et al., 2018). As detailed below, since m^1^G_37_ modification of several budding yeast tRNAs that are encoded by intron-containing genes require tRNA retrograde nuclear import, retrograde tRNA nuclear import must occur constitutively to generate functional tRNAs for protein synthesis (Figure 1). However, as accumulation of nuclear pools of tRNA from the cytoplasm also occurs upon stress conditions, the import process may also be inducible (Shaheen and Hopper, 2005; Takano et al., 2005, 2015; Hurto et al., 2007; Shaheen et al., 2007; Whitney et al., 2007). Two proteins from budding yeast have been identified as possible tRNA nuclear importers, Mtr10 (Shaheen and Hopper, 2005; Murthi et al., 2010) and Ssa2 (Takano et al., 2015); the latter is more likely to participate in regulated retrograde tRNA nuclear import (Figure 6).

#### Mtr10—A Ran-GTP Dependent Importer May Function in tRNA Nuclear Import

Budding yeast Mtr10 is a β-importin family member best characterized for its role in nuclear import of the protein Npl3 that is required for mRNA nuclear export (Senger et al., 1998). Mtr10 is also implicated in nuclear import of the TLC1 RNA subunit of telomerase that shuttles between the nucleus and the cytoplasm; cells lacking *MTR10* (*mtr10Δ*) have short telomeres, the levels of TLC1 are reduced and TLC1 does not normally accumulate in the nucleus. However, it is unknown whether Mtr10 is directly involved in TLC1 nuclear import (Ferrezuelo et al., 2002; Gallardo et al., 2008).

Three lines of evidence suggested that Mtr10 functions in tRNA retrograde nuclear import. First, tRNA nuclear import was reported to be dependent upon the Ran-GTP gradient, although this conclusion has been controversial (Shaheen and Hopper, 2005; Takano et al., 2005). Second, *mtr10Δ* cells fail to accumulate tRNA in the nucleus upon amino acid (aa) deprivation, in contrast to wild-type cells (Shaheen and Hopper, 2005). Third, *mtr10Δ los1Δ* mutants do not accumulate large nuclear pools of tRNAs (Murthi et al., 2010); as the level of nuclear accumulation in *los1Δ* cells is the result of defects in both tRNA primary nuclear export and re-export, reduced tRNA nuclear pools in the *mtr10Δ los1Δ* double mutant is most likely due to decreased import of tRNA from the cytoplasm. Despite these three lines of evidence, Mtr10 did not co-purify with tRNA under conditions in which Npl3 and Mtr10 interaction was readily detected (Huang and Hopper, 2015). Therefore, Mtr10 may affect tRNA nuclear pools indirectly. In vertebrate cells, the putative Mtr10 orthologue, TNPO3 (Transportin 3) serves to import serine-arginine-rich splicing factors into the nucleus (Maertens et al., 2014). No apparent role for TNPO3 in tRNA nuclear import has been detected; instead, it has been proposed that TNPO3 functions in disassembly of tRNA–capsid complexes from HIV pre-integration complexes after nuclear import (Zhou et al., 2011).

#### Ssa2—A Protein Chaperone Implicated in Ran-GTP Independent tRNA Nuclear Import

Takano et al. reported that retrograde tRNA nuclear import is ATP-dependent (Takano et al., 2005). Thus, to identify proteins that may participate in tRNA retrograde nuclear import, the Yoshihisa group searched for proteins from budding yeast able to bind tRNA in an ATP-sensitive fashion and thereby identified Ssa2. Ssa2 is a constitutively expressed chaperone and member of the heat shock protein 70 (HSP70) family with known functions in protein folding. However, members of the Hsp70 family also bind RNA AU-rich elements—sequences characteristic of unstable mRNAs (Henics et al., 1999; Takano et al., 2015) (Figure 6). Cells deleted for *SSA2* (*ssa2Δ*) fail to accumulate elevated tRNA nuclear pools when cells are deprived for aa (Takano et al., 2015). The results support a role for Ssa2 in nutrient-dependent regulated tRNA nuclear import. Ssa2 prefers unmodified to fully modified tRNA and it can bind a nuclear pore protein. Thus, Ssa2 may serve as a tRNA chaperone delivering defective tRNAs to the nucleus for quality control and as a cellular response to nutrient deprivation.

*ssa2Δ* cells that are also depleted for Mtr10 have significantly smaller nuclear pools of tRNA upon aa deprivation than either individual mutant, indicating that Ssa2 and Mtr10 independently affect tRNA retrograde nuclear import. The results provide evidence for both Ran-dependent and Ran-independent pathways for retrograde tRNA nuclear import and a role for each in interacting with multiple RNA and protein substrates (Figure 6).

#### tRNA Re-export From the Nucleus to the Cytoplasm

Data from budding yeast document that tRNAs imported from the cytoplasm to the nucleus *via* retrograde tRNA nuclear import are re-exported to the cytoplasm, provided that cells are provided with ample nutrients. First, cells acutely deprived of aa, glucose, or phosphate accumulate tRNAs in the nucleus; tRNA nuclear accumulation is not due to defects in primary tRNA nuclear export because the tRNAs are efficiently spliced under these conditions (Shaheen and Hopper, 2005; Hurto et al., 2007; Whitney et al., 2007; Huang and Hopper, 2014). Upon re-feeding with the appropriate nutrient, the tRNA nuclear pools rapidly dissipate (Whitney et al., 2007). Similarly, tRNA nuclear accumulation occurs in rat hepatoma cells upon aa deprivation, and upon re-feeding, there is rapid movement of the tRNAs to the cytoplasm (Shaheen et al., 2007).

Second, wybutosine (yW) modification of G_37_ of budding yeast tRNA^Phe^ requires both the primary tRNA nuclear export and re-export steps (Ohira and Suzuki, 2011). This is because the first step of yW modification is catalyzed by the Trm5 methyltransferase that acts only on spliced tRNAs but is located in the nucleus; so, tRNA^Phe^ must first be exported to the cytoplasm by the primary tRNA nuclear export process to be spliced on the surface of mitochondria and then imported back into the nucleus to gain m^1^G_37,_ catalyzed by Trm5. The subsequent steps of yW modification are catalyzed by cytoplasmic enzymes and therefore to complete yW modification, m^1^G_37_ modified tRNA^Phe^ must be re-exported to the cytoplasm (Ohira and Suzuki, 2011) (Figure 1). Similarly, queuosine (Q) modification of tRNA^Tyr^ in *T. brucei* requires that the pre-tRNA^Tyr^ be exported to the cytoplasm where its intron is removed; following import to the nucleus, the spliced tRNA is modified by nucleus-located tRNA-guanine transglycosylase, which has specificity for spliced tRNA^Tyr^. Q-modified tRNA^Tyr^ is then re-exported to the cytoplasm to fulfill its function in protein synthesis (Kessler et al., 2018).

Which exporters function in the tRNA re-export step? Evidence supports the model that Los1 and Mex67-Mtr2 that function in primary tRNA nuclear export, also function in tRNA re-export to the cytoplasm. As detailed above, the vertebrate and *S. pombe* Los1 homologues, Exportin-t and Xpot, are able to bind both intron-containing and mature tRNA; similarly in budding yeast, both unspliced and spliced tRNAs co-purify with Los1 (Huang and Hopper, 2015). Therefore, Los1 also serves in the re-export step to deliver spliced tRNA to the cytoplasm. Likewise, Mex67 co-purifies with spliced tRNA (Chatterjee et al., 2017), and therefore, it is likely to also function in tRNA nuclear re-export in budding yeast (Figure 5). In addition to Los1/Exportin-t and Mex67-Mtr2/NXF1-NXT1, another protein, Msn5/Exportin-5, functions in tRNA nuclear re-export.

#### Msn5/Exportin-5 - A β-importin That Participates in tRNA Nuclear Export in Yeast and Vertebrates

Yeast Msn5 and its exportin-5 vertebrate homologue have been shown to function in tRNA nuclear export. However, this β-importin family member serves additional roles in nuclear-cytoplasmic dynamics. Vertebrate and plant Msn5 homologues, Exportin-5 and Hasty (HST), respectively, serve to export pre-micro RNA (miRNA) from the nucleus to the cytoplasm (Yi et al., 2003; Bohnsack et al., 2004; Lund et al., 2004; Park et al., 2005; Kim et al., 2016). This is accomplished by direct binding of Exportin-t/HST to the hairpin structure of miRNAs and other RNAs (Shibata et al., 2006). In addition, vertebrate Exportin-5 functions in SRP RNA (Takeiwa et al., 2015) and adenoviral VA1 RNA (Gwizdek et al., 2003) nuclear export. Budding yeast Msn5 functions to export numerous proteins to the cytoplasm. These include the HO protein, that is required for mating type switching, and phosphorylated forms of transcription factors Pho4, Mig1, Crz1, and Maf1 that travel to the cytoplasm in response to various environmental conditions [Reviews: (Hopper, 1999; Ciesla and Boguta, 2008)]. Furthermore, the interaction between Msn5 and phosphorylated Pho4 and a peptide from HO are direct because binding occurs *in vitro* with purified components in the presence of Ran-GTP (Kaffman et al., 1998; Bakhrat et al., 2008) (Figure 5). There is no evidence showing binding of vertebrate Exportin-5 with proteins.

tRNAs have been shown to be aminoacylated in the nucleus in both vertebrate cells and budding yeast (Lund and Dahlberg, 1998; Sarkar et al., 1999; Grosshans et al., 2000; Azad et al., 2001), even though the vast majority of the aminoacyl tRNA synthetases reside and function in the cytoplasm for protein synthesis. Both in cultured vertebrate cells and in *Xenopus* oocytes, Exportin-5 was shown to promote nuclear export of aminoacylated tRNA (aa-tRNA) *via* a complex with translation elongation factor 1A (eEF1A) in a Ran-GTP-dependent mechanism (Bohnsack et al., 2002; Calado et al., 2002). The authors proposed that a major role for Exportin-5 in vertebrate cells is to prevent translation factors from accumulating in the nucleus. More recent studies document that SNAG-containing transcription factors can piggyback on the Exportin-5-aa-tRNA-eEF1A complex *via* eEF1A interaction so to provide a mechanism for nuclear export of this category of proteins (Mingot et al., 2013). Plant HST has no apparent role in tRNA nuclear export (Park et al., 2005).

The role of Msn5 in tRNA nuclear export in budding yeast was first demonstrated by FISH studies documenting tRNA nuclear accumulation in *msn5Δ* cells deprived for aa (Shaheen and Hopper, 2005; Murthi et al., 2010). However, as there is no defect in pre-tRNA splicing in *msn5Δ* cells, it was concluded that Msn5 does not participate in primary nuclear export for the 10 families of tRNAs that are encoded by intron-containing genes in budding yeast, but rather that Msn5 is dedicated to their tRNA nuclear re-export (Murthi et al., 2010). Studies to capture *in vivo* Msn5 with its tRNA cargo provide strong evidence that in a Ran-GTP-dependent mechanism, Msn5 interacts efficiently with spliced tRNA, but interacts very inefficiently with intron-containing tRNA (Huang and Hopper, 2015), supporting the selective role for Msn5 in the re-export of tRNAs encoded by intron-containing genes. Msn5 preferentially binds aa-tRNA in a quaternary complex with eEF1A and Ran-GTP (Figure 5).

The manner in which Msn5 assembles into a tRNA nuclear export complex provides explanations for earlier studies demonstrating roles for eEF1A, nuclear pools of aminoacyl synthetases, and the enzyme that adds CCA to tRNA 3′ termini (Cca1) in tRNA nuclear export (Sarkar et al., 1999; Grosshans et al., 2000; Azad et al., 2001; Feng and Hopper, 2002). Msn5 provides one mechanism for cells to regulate tRNA re-export in response to aa deprivation; it also serves to regulate the nuclear-cytoplasmic distribution of several transcription factors in response to environmental stress.

In summary, re-export of tRNAs in budding yeast employs at least three nuclear exporters: Los1, Mex67-Mtr2, and Msn5 (Figure 5). As Msn5 does not interact with intron-containing tRNA, it functions solely in the re-export step for the category of tRNAs encoded by intron-containing genes. As for the other transporters discussed, Msn5 has multiple different non-tRNA substrates and multitasks in their nuclear export.

### Function of RNA Subcellular Dynamics Between the Nucleus, Cytoplasm, and Mitochondria

Although discovery of the tRNA retrograde pathway was surprising and initially counter intuitive, subsequent studies document multiple functions for this complicated subcellular tRNA traffic. In budding yeast, tRNA retrograde dynamics function in translation for a subset of proteins (Chu and Hopper, 2013), in quality control of tRNA 5′ leader processing and modification [(Kramer and Hopper, 2013) Review: (Huang and Hopper, 2016)], and in addition of a particular tRNA nucleoside modification (Ohira and Suzuki, 2011). Other small RNAs, in particular, snRNA and TLC1 RNA, also shuttle between the nucleus to the cytoplasm and have been shown to have their processing steps occur both in the nucleus and the cytoplasm. So, RNA trafficking between the nucleus and the cytoplasm is not unique to tRNA. Furthermore, RNA processing on the surface of mitochondria is not restricted to pre-tRNA splicing (Chatterjee et al., 2018). In budding yeast cyclization of t^6^A_37_ to ct^6^A_37_ is catalyzed by Tcd1 and Tcd2 which are also located on the mitochondrial surface (Huh et al., 2003; Miyauchi et al., 2013). Likewise, Q_34_ tRNA modification catalyzed by the heteromeric transglycosylase in mouse occurs on the mitochondrial surface (Boland et al., 2009). Nor is RNA processing on mitochondria restricted to tRNA as the maturation of nucleases that catalyze processing of piRNA (PIWI-interacting RNA), 5′ Zucchini (MitoPLD) and/or 3′ trimmer (PARN-1), in mouse (Watanabe et al., 2011), flies (Huang et al., 2011), silk worm (Izumi et al., 2016) and *C. elegans* (Tang et al., 2016) reside on the mitochondrial cytoplasmic surface. Studies in budding and fission yeast documented a role for Tom70, a protein subunit of the TOM mitochondrial import complex, in localizing the SEN subunits to mitochondria (Wan and Hopper, 2018). It will be interesting to learn if Tom70 homologues in metazoans function to localize the other yeast and metazoan RNA processing enzymes to mitochondria.

## Epilogue

In stark contrast to the commonly held notion that separate gene products and mechanisms are employed to process different categories of RNAs, there are numerous examples of gene products or complexes involved in tRNA biology that multitask in the production and/or subcellular trafficking of other RNAs. Such multitasking likely arose as mechanisms for cells to streamline their genomes by having given gene products serve multiple tasks. There are other mechanisms in eukaryotic RNA biology that also have been selected to streamline genomes. For example, due to alternative starts of transcription and/or alternate translation starts, single genes encoding tRNA modification enzymes and aminoacyl synthetases generate multiple proteins with distinct subcellular locations so to deliver the same catalytic activities to separate destinations; for example, *TRM1* encodes two proteins, one targeted to mitochondria and the other to the nucleus and *CCA1* encodes three proteins that are targeted to the mitochondria, nucleus, or the cytoplasm [Reviews: (Martin and Hopper, 1994; Danpure, 1995)]. Moreover, some proteins in tRNA biology serve additional unrelated roles. For example, budding yeast Mod5 that catalyzes modification of A_37_ to i^6^A_37_ of particular tRNAs also functions in regulation of sterol biogenesis in the cytoplasm (Benko et al., 2000) and transcription silencing in the nucleus (Pratt-Hyatt et al., 2013) and numerous budding yeast and vertebrate tRNA aminoacyl synthetases possess functions independent of translation [Reviews: (Guo and Schimmel, 2013; Yakobov et al., 2018)]. Further exploration will undoubtedly uncover new examples of multitasking in the eukaryotic world of tRNA biology.

## Author Contributions

AH and RN each participated in the preparation of this review.

### Conflict of Interest Statement

The authors declare that the research was conducted in the absence of any commercial or financial relationships that could be construed as a potential conflict of interest.
